# Canadian Chicken-Cholera1Read at the Thirty-sixth Annual Meeting of the American Veterinary Medical Association, New York, September, 1899.

**Published:** 1899-10

**Authors:** Charles H. Higgins

**Affiliations:** Pathologist to the Department of Agriculture, Dominion of Canada


### THE JOURNAL

OF

### COMPARATIVE MEDICINE AND VETERINARY ARCHIVES.

VOL. XX. *OCTOBER, 1899.* No. 10.

### CANADIAN CHICKEN-CHOLERA.[1]

BY CHARLES H. HIGGINS, B.S., D.V.S.,

PATHOLOGIST TO THE DEPARTMENT OF AGRICULTURE, DOMINION OF CANADA.

(*From the Molson Pathological Laboratory of McGill University.*)

THE bacillus of fowl-cholera was first successfully cultivated in 1880 by Pasteur, whose investigations of this organism mark an epoch in the bacteriological study of disease, although it was previously observed by Perroncito and others. This bacillus, although well known and fully described, has not been identified as the causal factor of the disease upon this continent. Cholera in poultry, it is true, exists here, and in *Bulletin* No. 8 of the Bureau of Animal Industry there is to be found a very careful study of "Three Outbreaks of Fowl-cholera," by Dr. Veranus A. Moore. These epizoötics occurred in places far apart—one near Providence, R. I., another in Virginia, and yet another near Washington. The germ from all three proved to be the same. It differed, however, in its characters from the European bacillus of fowl-cholera, though closely allied thereto. The germ that I am about to describe would seem to resemble very closely, if indeed it is not identical with, the true bacillus of fowl-cholera as found in European countries—that is, it has been possible for me to isolate a germ which, it has been found, is very closely allied to one causing outbreaks of a similar nature along the Atlantic Coast, though distinct from this form, would seem sufficient ground for presenting a description of it and its characters.

This outbreak was brought to my notice in December, 1895, by Prof. Adami, who in turn had learned of it through Dr. G. P.

[1] Read at the Thirty-sixth Annual Meeting of the American Veterinary Medical Association, New York, September, 1899.

Girdwood. It occurred among a flock of fowls belonging to Mr. A., of St. Anne's, P. Q. St. Anne's is situated on the Island of Montreal, about twenty miles from the city, at the point where the St. Lawrence divides to encircle the island. I was enabled to visit the scene of the outbreak, where serious losses had occurred, procuring a number of the affected fowls.

*History.* About a month prior to my visit the fowls were dying very suddenly, without any apparent cause. The disastrous results were communicated to Mrs. G., among whose fowls there had been an outbreak of a similar nature several years previously. She suggested the placing of sulphuric acid and sulphate of iron in the drinking-water, and allowing access to no other water; also complete disinfection of the hen-house. After the administration of the above remedy, together with a cleansing of the quarters, the disastrous results were checked, few fowls dying subsequently. The disease pursued a very rapid course, producing death in from three to twelve hours from the time that the first symptoms were noticed. The greater number were found dead in the morning (from two to four at a time). The total loss was about twenty, which were invariably the youngest. The older ones seemed immune, and none showed symptoms of the disease.

*Symptoms.* There was a profuse diarrhœa of a yellow color, the feathers were ruffled, and there was yellowness about the head. They were not inclined to move after the appearance of noticeable symptoms, but rather rolled themselves up into a ball, so to speak, remaining motionless till they fell over dead.

The hygienic conditions were favorable to the development of any infectious disease. The place was small and damp, with very little light and little or no ventilation. After the outbreak a fire was started in order that the apartment might be thoroughly dried.

Three fowls dead of the disease were procured. It was not definitely known how long they had been dead, but it was thought to have been at least a week, as they had been thrown outside upon being found, and were at the time of visit covered with snow. For several days it had been cold; there had occurred five days previously a snowstorm, and another the day previous to my visit. To all appearances the fowls were covered with both falls of snow. Probably they had been exposed during the time that they had lain outside to a mean temperature of at least — 20° C., it having been very cold after the first fall of snow. On the night previous to their being carried to the laboratory they were left in a sleigh out of doors, a thermometer nearby registering — 27° C.

*Morbid Anatomy.* The appearance found at the autopsy will be cited in but one case, as there was little or no difference noted in the three. The subject was a mongrel Plymouth Rock hen, about eight months old. She was in good condition. The liver was pale, with areas of necrosis; spleen soft, pulpy, and enlarged; the intestines pale externally and free from evidence of hemorrhage; within the mucosa was congested and swollen; lower portion of the intestine particularly affected and its follicles moderately distinct; there was fluid in the pericardial cavity, with some hemorrhage beneath the pericardium and marked bloody imbibition of the vessel-walls of the various organs.

Cultures were made from the various organs and fluids of the body, and revealed on the following day a pure and moderately vigorous growth of a non-motile bacillus. The germs from the various cultures were found to be identical and pure.

### *Description of the Bacillus.*

*Morphology.* The microörganism is a non-motile, rod-shaped organism, aërobic, though growing in the presence of a little free oxygen, varying slightly in size according to the medium upon which it is grown. In broth at 37° C., at the end of twenty hours, the bacilli vary from 1.3$\mu$ to 1.8$\mu$ in length and from 0.5$\mu$ to 0.7$\mu$ in breadth. The most frequent form is a diplobacillus with rounded ends, which may under low magnification be mistaken for a diplococcus. In hanging-drop preparations it shows a distinct polar arrangement of the protoplasm. It is frequently seen in old cultures in short chains, consisting of three or four of the bacilli placed end to end. It stains with the ordinary anilin dyes, but retains the coloring-matter feebly or not at all when subjected to Gram's method. Great numbers are seen in the tissues and blood, often occurring in clumps of four or five. No spores or tendency to spore-formation have been recognized. A feebly staining capsule has been occasionally observed.

*Cultures in Broth.* This medium was made by using 2 grammes of Liebig's extract to 500 c.c. of distilled water, 1 per cent. of Witte's peptone, and 0.5 per cent. rock-salt. Kept at 37° C. the broth is uniformly clouded at the end of twenty-four hours. This cloudiness does not, as a rule, settle, but in some cases the broth eventually cleared, the conditions remaining the same. The bacilli were still alive in tubes which had been inoculated nine weeks. They were also alive in sealed tubes at the end of one year, but were not alive at the end of three years. It grows equally well in acid and alkaline media within certain limits. Fermentation experiments with the various sugars proved negative.

*Nutrient Gelatin Cultures.* Here the Liebig extract broth was used, 7 per cent. of gelatin being added. The growth in this medium is less vigorous, and is recognizable about the fourth day. In stab-cultures there is little growth on the surface, being equal all along the line of inoculation. On plates, superficial colonies about 0.5 mm. in diameter are seen on the third or fourth day; the deep colonies are more minute. The surface growths are granular, without sharply defined borders. The deep colonies show a granular appearance; their color is a pale white. There is no liquefaction. It was noticeable that no cultures were obtained beneath mica plates. In other words, the germ will not grow when deprived of free oxygen, but grows better where there is little oxygen (along stabs) than where oxygen is abundant (on the surface).

*Agar Cultures.* The Liebig broth was used for this purpose, 1 per cent. agar being added. Growth on this medium at 37° C. is not vigorous at the end of twenty-four hours; a pale white, almost colorless streak, of glistening appearance, appears along the line of inoculation. Isolated colonies are about 0.2 mm. to 0.5 mm. in diameter, convex, with granular edges. Acid is produced, but not in abundance. Lactose-litmus-agar shows a red coloration along the streak on the second day. There is a reddening of the surrounding medium only after the fourth day, which is not extensive. From plates and tubes there arises a peculiar penetrating odor.

*Potato Cultures.* A slight yellowish coloration appears after the fourth day, but it is not vigorous.

*Milk.* A change in the consistency of the cream is noted after the fourth day. No other change is noted after six weeks in the incubator.

*Blood-Serum.* The natural blood-serum from cattle, coagulated, was used. The growth is very similar to that obtained on agar, but is not nearly so vigorous. The growth is seen along the line of inoculation, broadening if kept in the incubator for a time.

*Temperature Relationships.* The germ will grow at the ordinary room-temperature and also at that of the blood. A temperature of 59° C. did not destroy the organism in ten minutes, but one of 60° C. for the same length of time destroyed its vitality. It has already been pointed out that the germ is able to resist a temperature lower than 0° C. for several days, and one of — 27° C. for several hours, although this temperature probably leads to some attenuation.

*Action of Disinfectants.* A 1 per cent. solution of carbolic acid destroyed the life of the germ in five minutes. A 0.5 per cent. solution of hydrochloric acid was fatal in the same length of time, as was also a 0.5 per cent. solution of sulphuric acid. For these latter experiments I used the commercial sulphuric and hydrochloric acids.

*Results of Inoculation.*

*Poultry.* A fowl inoculated intravenously with 2 c.c. of a twenty-four-hour-old culture was found dead on the second day. Further than this the virulence for fowls has not been determined, owing to an accident in which the virulent germ was lost. Following the inoculation the fowls were listless, and there was a profuse diarrhœa noted even where the attenuated germ was used, the stools consisting of a semi-mucoid mass, greenish-yellow or white in color.

The autopsy showed the intestines filled with gas, crop empty, gizzard containing small stones and ingesta. The intestines contained a greenish-yellow, semi-mucoid mass. Kidneys normal. Spleen congested and enlarged. Liver fatty; gall-bladder much distended. Heart and lungs normal. Where the fowls had been given a subcutaneous injection of a less virulent form into the breast-muscle there was at this point a mass of cartilaginous hardness, yellow in color, free in the muscle-tissue. The bacilli were found in all the fluids and tissues of the body. Immunity has been produced in young chickens by the injection of a moderate quantity of an attenuated virus.[1]

*Turkey.* A turkey was inoculated intravenously with 6 c.c. of a twenty-four-hour-old culture, symptoms appearing about an hour afterward, at which time he was loath to stand; the feathers were slightly ruffled and the head drawn close to the body. Further than this the symptoms were not noticed, as the fowl was inoculated in the afternoon and found dead in the morning, in the same spot, having succumbed in about fifteen hours.

The autopsy, made a few hours after death, revealed the following: Liver, great congestion, otherwise normal. Heart, lungs, spleen, and kidneys normal. The intestines were pale externally, but from within the mucosa was seen to be swollen and greatly congested, showing ecchymoses and areas of necrosis. The congestion was not localized, but extended from the mouth to the

[1] There would seem to have been a very definite attenuation of the bacilli owing to the fact that they were obtained from the bodies of fowls that had been dead for some time, being preserved by the extreme low temperature, which was far below 0° C.

anus. The intestine was filled with partially digested food of a greenish color. The crop was greatly distended with ingesta.

*Rabbits.* The first experimental rabbit was inoculated by the ingestion of bacilli upon food, dying on the twelfth day. The course followed by the disease was that of a septicæmia, the symptoms of which were characteristic. The next inoculation was with a twenty-four-hour-old culture from the first animal, death occurring in two days. From this time the germ rapidly gained virulence as it was passed from one to another, the last, which was the ninth of the series, dying six hours after inoculation. This last animal was inoculated with 3 c.c. of a preparation of ten drops of blood from the previous animal and 10 c.c. of broth.

The autopsy presented the following lesions: Upon opening the abdominal cavity an acute peritonitis was noted, with an effusion of serum. The intestines were distended with gas. The mucosa of the stomach peeled off with ease, the surface being congested and studded with ecchymoses. The intestines contained a greenish-yellow, semi-mucoid mass, and were injected. Appendix discolored; mucosa congested with small ecchymoses. Liver firm; gall-bladder greatly distended. Kidney slightly congested. Heart and lungs normal. Congestion, with partial necrosis, was noted on the mucosa of the uterus. Bacilli were found in the blood, tissues, and cultures from the various organs. It was in this particular case demonstrated in the vitreous humor of the eye.

Marked symptoms were seen in the inoculation disease in rabbits. There was drowsiness, the hind-legs were drawn well under the body, and the fore-legs forced back as far as possible. With the virulent germ there was a profuse diarrhœa, appearing a few hours after inoculation and in those animals withstanding for some time tubercle-like masses were found in the appendix. Inoculated with the most virulent virus, the acute form of the disease developed, and they remained motionless until death supervened.

A *guinea-pig* inoculated with 1 c.c. of a broth culture, inside the thigh, developed a local abscess.

*Dogs* fed on dead subjects developed no marked symptoms save a profuse diarrhœa. There was no elevation of the temperature.

This bacillus corresponds closely with the European germ of chicken-cholera in all respects. So close is the resemblance that I cannot but hold that the two are identical, and that I have been dealing with the true disease, being the first epizoötic in America. Considering the statements of previous observers, I have found divergences in but two respects, namely, the resistance of the organism to heat and disinfectants. Baumgarten states that the European germ is destroyed by an exposure of fifteen minutes to 55° C., or, according to Salmon, by an exposure of ten minutes to 56° C. The form here described was killed by a temperature of 60° C. after a ten minutes' exposure. It appears to be more sensitive to disinfectants, for, according to Heuppe, the European form was killed by 3 per cent. carbolic acid in six hours. The Canadian form did not grow after being left in a 1 per cent. carbolic solution for five minutes. These discrepancies are considerable, but we must remember that the want of agreement in the results of various observers in connection with these two tests is notorious, and that when these two tests alone yield divergences we have not sufficient ground for stating that we are dealing with a different species.

There is a distinct separation between this form and that already recognized in America. The Canadian germ is smaller; takes the polar stain well; does not saponify milk; produces acid in dextrose and lactose broths; can be rendered most virulent for rabbits, a drop or two of the blood being sufficient to kill in six hours, and in both rabbits and fowls there is developed a most profuse diarrhœa. I have found, in addition to these differences, that the American form is much more vigorous in its growth on gelatin (both stab and streak cultures), being easily recognizable in twenty-four hours, while the Canadian and European forms show nothing till the fourth day. The colonies of the American form on plate cultures are much smaller, with much more sharply defined borders. There is little or no difference upon agar.

The study of the whole of the large group of bacilli of hemorrhagic septicæmia has revealed so much variability, so many individual points of difference between microbes in the main closely resembling each other, that I shall not venture to say that the forms isolated by Dr. Moore belong to a distinct species. I only urge that the Canadian form approaches more nearly the classical type.

Successfully checking the ravages of this disease is a comparatively easy matter by strict attention to the sanitary surroundings, the germ being particularly susceptible to the action of heat and the various antiseptics.

---

Dr. J. W. Dunham, of Dakota, has removed to Fargo, North Dakota, where he will continue the practice of his profession.

